# Synthesis and Sensing Response of Magnesium Antimoniate Oxide (MgSb_2_O_6_) in the Presence of Propane Atmospheres at Different Operating Voltages

**DOI:** 10.3390/s24072147

**Published:** 2024-03-27

**Authors:** Héctor Guillén-Bonilla, José Trinidad Guillén-Bonilla, Verónica-María Rodríguez-Betancourtt, Jorge Alberto Ramírez-Ortega, Juan Pablo Morán Lázaro, Alex Guillén-Bonilla

**Affiliations:** 1 Departament of Project Engineer, CUCEI, Universidad de Guadalajara, M. García Barragán 1421, Guadalajara 44410, Mexico; hector.guillen1775@academicos.udg.mx; 2 Departament of Electro-Photonics, CUCEI, Universidad de Guadalajara, M. García Barragán 1421, Guadalajara 44410, Mexico; trinidad.guillen@academicos.udg.mx; 3 Departament of Physics, CUCEI, Universidad de Guadalajara, Guadalajara 44410, Mexico; veronica.rbetancourtt@academicos.udg.mx (V.-M.R.-B.); jorge.rortega@academicos.udg.mx (J.A.R.-O.); 4 Department of Computer Science and Engineering, CUVALLES, Universidad de Guadalajara, Carretera Guadalajara-Ameca Km 45.5, Ameca 46600, Mexico; pablo.moran@academicos.udg.mx

**Keywords:** MgSb_2_O_6_, thick films, sensitivity, gas sensor

## Abstract

Nanoparticles of MgSb_2_O_6_ were synthesized using a microwave-assisted wet chemistry method, followed by calcination at 700 °C. Their ability to detect different concentrations of propane gas (C_3_H_8_) at various operating voltages was evaluated. The material’s crystalline phase was identified using X-ray powder diffraction (XRD). The morphology was analyzed by scanning electron microscopy (SEM), finding bar- and polyhedron-type geometries. Through transmission electron microscopy (TEM), we found particle sizes of 8.87–99.85 nm with an average of ~27.63 nm. Employing ultraviolet–visible (UV-Vis) spectroscopy, we found a band gap value of ~3.86 eV. Thick films made with MgSb_2_O_6_ powders were exposed to atmospheres containing 150, 300, 400, and 600 ppm of propane gas for dynamic testing. The time-dependent sensitivities were ~61.09, ~88.80, ~97.65, and ~112.81%. In addition, tests were carried out at different operating voltages (5–50 V), finding very short response and recovery times (~57.25 and ~18.45 s, respectively) at 50 V. The excellent dynamic response of the MgSb_2_O_6_ is attributed mainly to the synthesis method because it was possible to obtain nanometric-sized particles. Our results show that the trirutile-type oxide MgSb_2_O_6_ possesses the ability, efficiency, and thermal stability to be applied as a gas sensor for propane.

## 1. Introduction

Extensive research is being conducted on semiconductor materials for their potential use as gas sensors [1]. These materials are known for their exceptional ability to change their electrical resistance when subjected to varying concentrations of certain gases (such as oxidants or toxins) in the presence of oxygen [1,2]. The electrical response of these semiconductors is based on the adsorption and desorption of oxygen ions on the surface [3,4] and the mobility of the charge carriers (holes or electrons) due to the temperature employed in the tests [1,2,3,4,5,6]. The variation in the electrical signal (resistance) shown by the semiconductor oxides depends on the detected gas and the type of semiconductor (p or n) [6,7]. In the gas sensor field, n-type semiconductors are the most studied [8] because they show excellent electrical response, thermal stability, and efficiency toward almost any gas (CO, O_2_, CO_2_, SO_2_, CH_4_, etc.) [8,9]. In addition, the advantages of using semiconductor oxides (whether p- or n-type) as gas sensors lie in the low production cost, the ease of use, the high capacity to detect various gases, and the simple manufacture of detection devices [7,8,10]. According to the literature, the ideal semiconductors for gas detection allow evaluation of their capacity and performance through parameters such as sensitivity, selectivity, response time, reversibility, and recovery time [11]. These parameters are strongly related to the material’s microstructural features, such as particle size, agglomeration, morphology, surface area, and porosity [12]. Other aspects that affect the detection properties are temperature, the thickness of the sensor surface [12], and its shape (which can be thin or thick films or pellets) [13,14,15]. Such features improve performance and efficiency in detecting low and high concentrations of gases. 

Since the ability of a semiconductor to detect gases was discovered [8,13], n-type binary oxides (such as ZnO and SnO_2_ [16]) have been the most studied materials due to their fast response, high sensitivity, thermal stability, selectivity, reproducibility, short response and recovery times, and high reliability [8,16,17]. Other p- and n-type binary oxides have also been intensively studied for their potential application as gas detectors [1]. Among them are: TiO_2_, WO_3_, α-Fe_2_O_3_, In_2_O_3_, Cu_2_O, Co_3_O_4_, Cr_2_O_3_, Mn_3_O_4_, and NiO [4,17]. Some authors have reported that the ternary semiconductors LaCoO_3_ [18], NdCoO_3_ [19], ZnAl_2_O_4_ [1], ZnMn_2_O_4_ [20], and CoTa_2_O_6_ [21] can be considered for use as gas sensors due to their good electrical response and thermal stability in different atmospheres. Additionally, several research groups have found that antimonates with different divalent cations (with formula MSb_2_O_6_, where M can be any cation of Ni, Co, Zn, Mn, etc.) could be used as gas sensors [7,22]. Some of the antimonates that have been studied include CoSb_2_O_6_ [22,23,24], MnSb_2_O_6_ [25], and ZnSb_2_O_6_ [26]. At room temperature, CobSb_2_O_6_ showed an excellent response to liquefied petroleum gas (LPG) concentrations of 5000 ppm [22]. Similarly, MnSb_2_O_6_ detected propane levels from 50 ppm at 100 °C [25], while ZnSb_2_O_6_ demonstrated good sensitivity to LPG concentrations from 5000 ppm. According to a recent study [7], nickel antimonate (NiSb_2_O_6_) showed an excellent response in static propane atmospheres, which led to the development of a gas detection prototype based on pellets of the oxide. These studies found that materials with a trirutile-type structure have good response and recovery times, stability, and reproducibility in propane atmospheres.

In general, the gases in which trirutile-type antimoniates have been tested the most are O_2_, CO, CO_2_, C_3_H_8_, and LPG [22,23,25,26], showing excellent electrical response and thermal stability, which is attributed to the morphology, as well as the nanometric particle size obtained when the oxides are synthesized [14,26]. As mentioned above, the ability, efficiency, and stability of gas sensors are closely related to the nanometric particle size of the semiconductor material [11]. This is because when the particle size is between 1 and 100 nm, the specific surface area increases, favoring the improvement in the catalytic activity to adsorb gases on the semiconductor’s surface and the electrical response [2,11,27]. Studies have shown that various metallic semiconductor nanostructures such as nanowires, nanotubes, core–shells, nanofibers, nanoflowers, nanosheets, and random figured nanoparticles [2,18] exhibit lower operating temperatures, increased sensitivity, improved selectivity, and lower response and recovery times [27]. In a previous study, we synthesized magnesium antimonate (MgSb_2_O_6_) nanorods to measure its detection properties in static CO and C_3_H_8_ atmospheres, achieving high sensitivity (~245.75 and ~61.66, respectively) when increasing gas concentration and operating temperature (from 23 to 300 °C) [28]. 

In this work, we synthesized magnesium antimonate by applying a wet chemistry process assisted with microwave radiation. This method is simple and cheap and allows good control of the material’s microstructure. Thick films were fabricated with powders of the oxide to study its ability to detect propane atmospheres at different concentrations, voltages, and operating temperatures. The material showed high sensitivity, thermal stability, efficiency, and reproducibility, which are desirable features of a gas sensor.

## 2. Materials and Methods

### 2.1. Synthesis

The wet chemistry method reported by Casillas-Zamora et al. [25] was used to synthesize MgSb_2_O_6_ nanoparticles with a trirutile-type structure. The reactants were magnesium nitrate hexahydrate (Mg(NO_3_)_2_·6H_2_O, Sigma-Aldrich, Jalmek, Guadalajara, Mexico, 99%), antimony trichloride (SbCl_3_, Sigma-Aldrich, Jalmek, Guadalajara, Mexico, 99%), ethylenediamine (C_2_H_8_N_2_, Sigma-Aldrich, 99%), and ethyl alcohol (C_2_H_5_OH, Jalmek, Guadalajara, Mexico, 99.5%). Three solutions were prepared using 1.28 g of Mg(NO_3_)_2_·6H_2_O, 2.80 g of SbCl_3_, and 5 mL of ethylenediamine separately. To each solution, 5 mL of ethyl alcohol was added, except for the C_2_H_8_N_2_ solution, to which 10 mL of the alcohol was added. All solutions were left under constant stirring at 300 rpm for 20 min at 25 °C. Then, the Mg(NO_3_)_2_·6H_2_O and C_2_H_8_N_2_ solutions were mixed. During the synthesis, the two amino groups forming ethylenediamine’s structure favored the formation of metal complexes by capturing the Mg^2+^ ions with the nitrogen’s free electrons, generating a large molecular mesh. Next, that solution was mixed with the SbCl_3_ solution, which joined the ethylenediamine-Mg^2+^ metal complex to form the Mg_2_Sb_2_O_6_. The final mixture was kept under constant stirring for 24 h at 25 °C. After the stirring, the ethyl alcohol was evaporated by applying microwave radiation (18 irradiations of 60 s) in a domestic oven (General Electric, model JES769WK) at a power of 140 W. The total energy applied to the solution was 151.2 kJ. The purpose of applying microwave radiation in steps of 60 s was to maintain the colloidal solution below 70 °C and thus avoid material loss due to splashes. After evaporation, the resulting material was dried at 200 °C for 8 h and then calcined at 700 °C in a Novatech muffle. The heating rate to reach 700 °C was 100 °C/h. Then, it was left at that temperature for 5 h.

### 2.2. Physical Characterization 

To analyze the crystalline structure of the oxide calcined at 700 °C, a diffractometer (Panalytical Empyrean, Guadalajara, Mexico) coupled to a deuterium/tungsten-halogen lamp was used, applying Cuα radiation with a wavelength (λ) of 1.5406 Å and a continuous scan (2θ) from 10 to 90° using 0.026 ° steps at a rate of 1 s/step. The value of the semiconductor’s forbidden bandgap was found by UV-Vis–NIR spectroscopy (UV-3600 Plus, Mexico City, Mexico). The absorbance spectrum was taken in a shift range from 200 to 700 nm. The calcined oxide’s microstructure was analyzed by field-emission scanning electron microscopy (FE-SEM) using a Tescan MIRA 3 LMU (Mexico City, Mexico) system with an acceleration voltage of 10 kV in a high vacuum. Transmission electron microscopy (TEM) was employed to study nanoparticles’ morphology and size using a Jeol system (model JEM-2010, Mexico City, Mexico) with an acceleration voltage of 100 kV. For this, the MgSb_2_O_6_ powders were dispersed in alcohol by ultrasound and drop-deposited onto Formvar-coated microgrids.

### 2.3. Dynamic Tests in C_3_H_8_ Atmospheres

**Figure 1:**
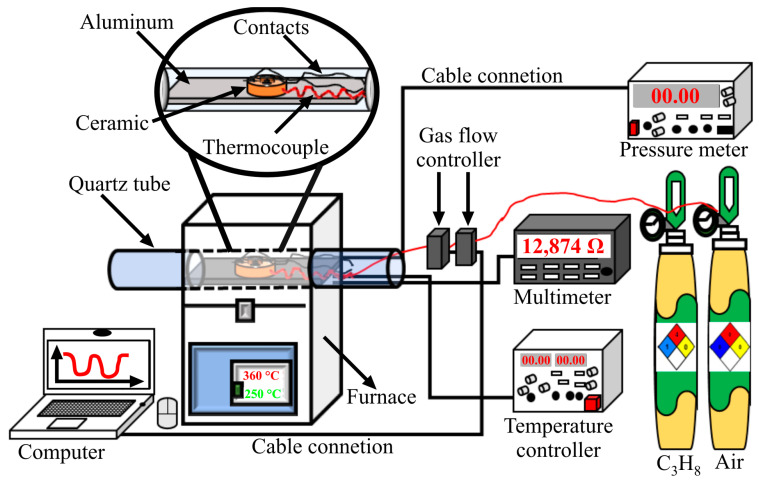
Schematic representation of the system used in the dynamic tests in air–C_3_H_8_ flows at controlled concentrations and temperatures.

Dynamic tests were carried out in air–C_3_H_8_ flows using thick films manufactured with powders of the MgSb_2_O_6_ calcined at 700 °C. A ceramic base with a central circular cavity and four ceramic millicolumns around the cavity was used. Each millicolumn had a small hole at half its length through which high-purity platinum wires (0.006 in diameter) were inserted to form the electrodes connected to the detection system. Then, 0.4 g of MgSb_2_O_6_ was dispersed in ethyl alcohol and placed dropwise into the ceramic base’s cavity to form a film ~500 µm thick and ~300 µm in diameter. Subsequently, the thick film was dried at 300 °C using a heating ramp of 100 °C/h for 4 h in a programmable muffle (Vulcan, model 5–550). The device was then placed in a quartz tube and inside a tubular furnace (Lindberg/blue) with programmable temperature control (Figure 1). The concentration of the air–C_3_H_8_ flows was controlled utilizing Brooks Instruments’, Cleveland, OH, USA, GF100CXXC-SH452.6L (2600 cm^3^/min) and GF100CXXC-SH40010C (10 cm^3^/min) mass flow regulators. When the material was exposed to the gas flows, the variation of its electrical resistance was quantified using a multimeter. The gas detection system was controlled with LabView 8.6 software (National Instruments, Cleveland, OH, USA). A schematic of the system used for the experiments is depicted in Figure 1. 

## 3. Results

### 3.1. XRD Analysis

**Figure 2:**
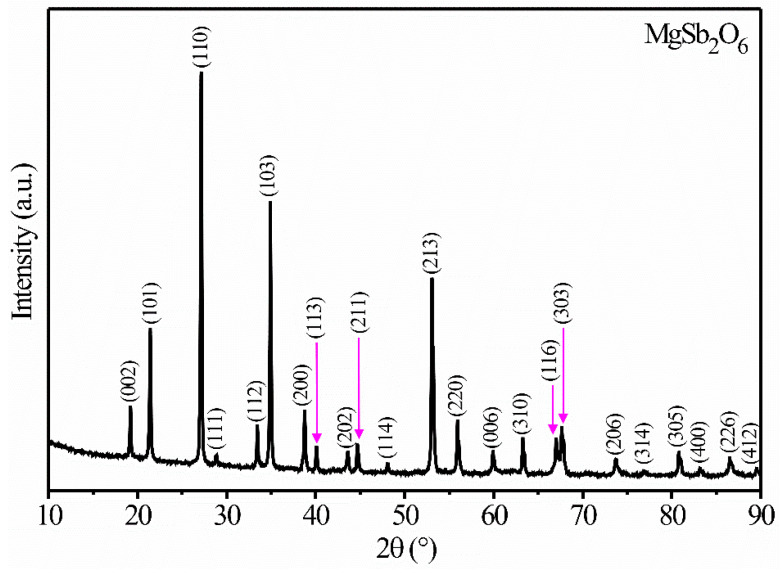
Diffractogram of powders of the MgSb_2_O_6_ calcined at 700 °C in air.

Figure 2 shows the diffractogram of the MgSb_2_O_6_ calcined at 700 °C. Using PDF file No. 88-1725, it was possible to identify the peaks associated with the oxide’s phase at 2θ = 19.17°, 21.41°, 27.15°, 28.85°, 33.46°, 34.91°, 38.78°, 40.10°, 43.61°, 44.72°, 48.10°, 53.16°, 56.01°, 59.95°, 63.33°, 67.06°, 67.72°, 73.73°, 76.78°, 80.87°, 83.22°, 86.57°, and 89.55°, corresponding to the crystalline planes (002), (101), (110), (111), (112), (103), (200), (113), (202), (211), (114), (213), (220), (006), (310), (116), (303), (206), (314), (305), (400), (226), and (412), respectively. According to that, the MgSb_2_O_6_ belongs to the family of trirutile-type materials, with a tetragonal crystalline structure, cell parameters a = 4.64 Å and c = 9.25 Å, and a space group P42/mnm (136) [28,29]. High crystallinity and purity are also inferred from the diffractogram, as reported in other works [25,29,30]. To determine the crystallite size, Scherrer’s equation [24] was used:

$$t=\frac{0.9\lambda }{\beta cos\theta },$$

where *λ* is the wavelength (1.5406 Å), *β* is the full width at half maximum of Bragg’s peak, and *θ* is Bragg’s angle. The most intense peak (110) corresponding to the last calcination was considered for the calculation. A crystallite size of ~43.48 nm was obtained.

Comparing our results (shown in Figure 2) with the literature, we confirmed that the MgSb_2_O_6_’s crystalline phase was obtained without secondary phases by the heat treatment (at 700 °C for 5 h). Other authors synthesized MgSb_2_O_6_ using the solid-state reaction method, subjecting the powder to 1000 °C for 48 h [29]. Nagarajan and Naraginti [30] reported that the oxide was synthesized by the solid-state reaction, calcining at 600 and 900 °C for 12 and 6 h, respectively. In a previous study, we synthesized MgSb_2_O_6_ nanoparticles at 800 °C using a chemical method, identifying a secondary phase associated with carbon (C) [28]. Thus, the synthesis route and the applied calcination temperature employed in this work are the best method to obtain MgSb_2_O_6_’s crystalline phase.

### 3.2. UV-Vis Analysis

**Figure 3:**
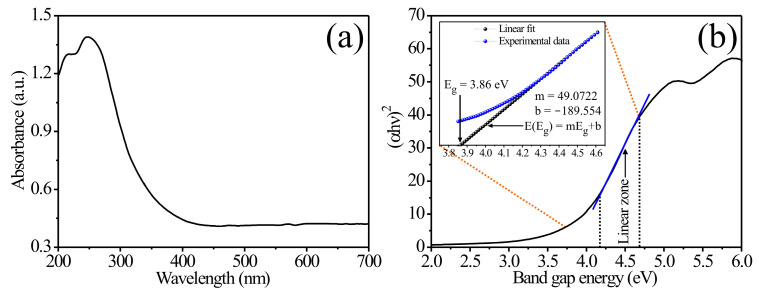
(**a**) A characteristic spectrum of the MgSb_2_O_6_, (**b**) value of the oxide’s forbidden band.

A UV-Vis absorption spectrum of the MgSb_2_O_6_ calcined at 700 °C is shown in Figure 3a, measured in a wavelength range of 200 to 700 nm (2.00 to 6.00 eV). In the 200–300 nm range, bands characteristic of materials with a trirutile-type structure [30,31,32,33] were identified. To evaluate the MgSb_2_O_6_’s forbidden energy bandgap, Tauc’s formula was used: ${\left(\alpha h\upsilon \right)}^{n}=A\left(hv-E_{g}\right)$, where α is the absorption coefficient, hν is the discrete energy, *A* is the band parameter, $E_{g}$ is the band’s energy gap, and *n* depends on the semiconductor transition type, where *n* = 2 for the direct transition [30,31,32]. The estimated value was ~3.86 eV (Figure 3b). 

This result is consistent with previous studies [31,32]. For example, Arunkumar and Naraginti [31] found that when exchanging the divalent cation of the trirutile structure (CoSb_2_O_6_, CuSb_2_O_6_, NiSb_2_O_6_, and FeSb_2_O_6_), the bandgap value ranged from 2.10–3.83 eV. Nagarajan and Naraginti [30] obtained values in the range of 3.30–4.05 eV for the MgSb_2_O_6_. Our value of ~3.86 eV is within those reported for trirutile-type semiconductors [30,31]. The band gap value in a trirutile-type semiconductor is strongly related to the synthesis method and the cation incorporated into the crystal lattice [2,17,31]. 

### 3.3. SEM Analysis

**Figure 4:**
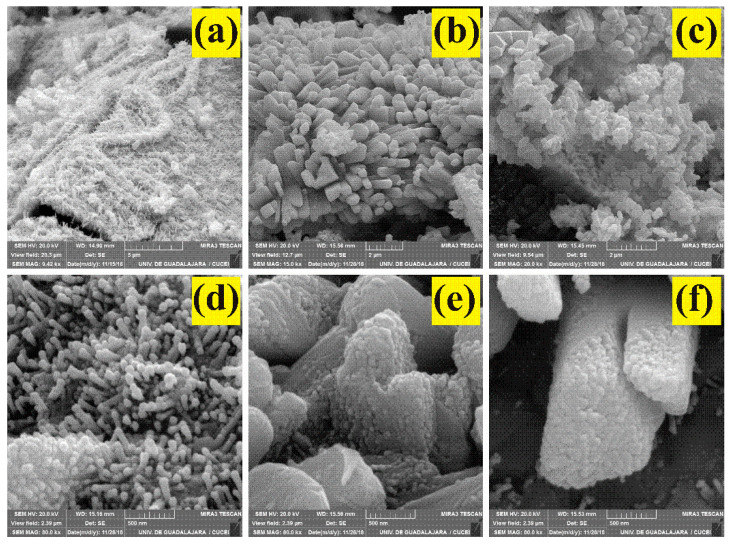
SEM images of powders of the MgSb_2_O_6_ calcined at 700 °C at magnifications of: (**a**) 9.42 kx, (**b**) 15 kx, (**c**) 20.00 kx, (**d**) 80 kx, (**e**) 80 kx, (**f**) 80 kx.

Figure 4 depicts six typical photomicrographs at magnifications of 9.42 kx 15 kx, 20.00 kx, 80 kx, 80 kx, and 80 kx of the microstructure of the MgSb_2_O_6_ calcined at 700 °C. Figure 4a shows that the oxide’s surface comprised very fine filament-type particles. Some appear like different-sized microneedles evenly distributed throughout the material’s surface. Figure 4b–d show polyhedron- and bar-shaped particles composed of smaller particles of different sizes (~0.1 μm). Figure 4e,f depict the growth in all directions of rods made up of assembled smaller particles (average size ~90 nm). It is worth mentioning that the particles agglomerated on the entire surface due to the material’s heat treatment.

**Figure 5:**
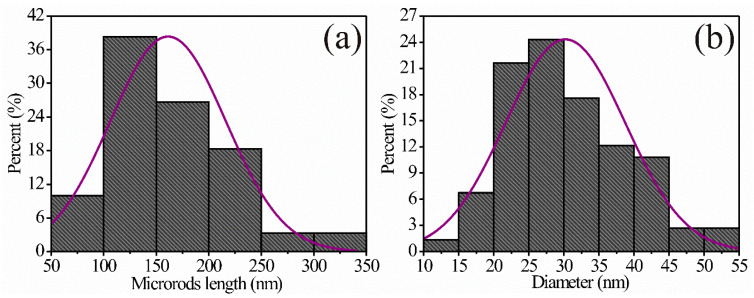
Distribution of (**a**) length and (**b**) diameter of microrods of the MgSb_2_O_6_ calcined at 700 °C.

To estimate the length and diameter of the microrods, several SEM images of different areas of the surface were required (Figure 5). The average size of the microrods was calculated in the range of 50–350 nm, with a mean of ~161.31 nm and a standard deviation of ~±54.48 nm (Figure 5a). The diameter of the microrods was calculated in a range of 10–55 nm, with a mean of ~30.22 nm and a standard deviation of ~±8.54 nm (Figure 5b). The measurements were carried out where the particles were clearly identifiable.

According to Figure 4, the formation of microrods, microneedles, and other similar morphologies is strongly related to the synthesis method [2,10,11,18]. Lamer and Dinegar proposed a possible mechanism for the nucleation and growth of particles, like those obtained here, using chemical methods. The rationale lies in increasing species’ concentration quickly until reaching a critical concentration. As a result, nuclei begin to form until a supersaturation concentration is reached. The nucleation process will end, and the particle growth will continue until the solubility reaches an equilibrium [34]. One of the drawbacks of Lamer and Dinegar’s model is the redissolution and precipitation of smaller particles (“Ostwald ripening”). To remedy that, the use of organic complexes such as ethylenediamine (considered as a stabilizer), surfactant molecules, or bases with nanometric structures that help maintain greater control in the particles’ nucleation and growth processes has been proposed [35,36], which gives rise to the formation of polyhedral and bar structures, such as those obtained in this work. 

### 3.4. TEM Analysis

**Figure 6:**
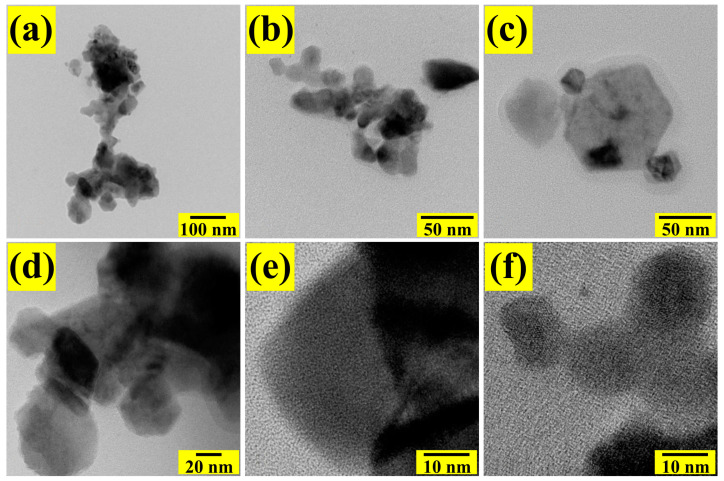
TEM images showing the nanostructured morphologies of the MgSb_2_O_6_ powders calcined at 700 °C: (**a**,**b**) agglomerated particles, (**c**,**d**) hexagonal morphology and (**e**,**f**) agglomerated polyhedral morphology.

**Figure 7:**
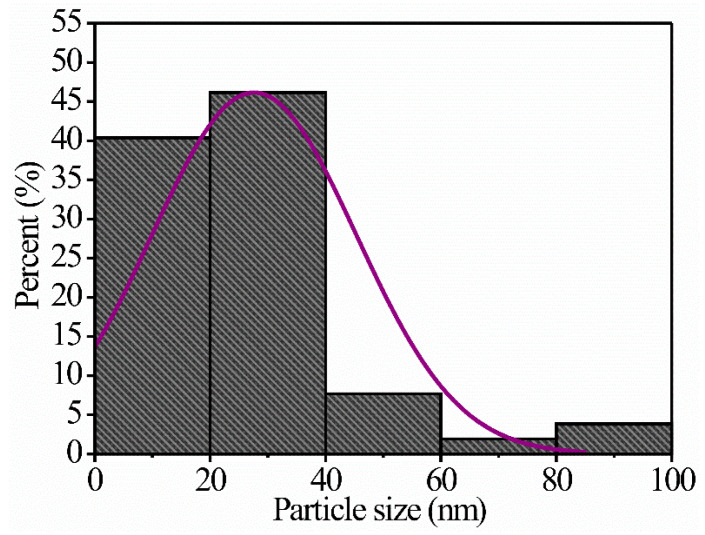
Particle size distribution of the MgSb_2_O_6_ calcined at 700 °C.

Figure 6 shows typical TEM images of the microstructure of the MgSb_2_O_6_ powders calcined at 700 °C. It is essential to mention that the powders were previously dispersed in ethyl alcohol using a sonifier to be able to analyze the particles individually. In Figure 6a–c, an agglomeration of differently sized particles (in the order of nanometers) is observed. These nanoparticles are linked together by necks formed by the heat treatment, the coalescence of the particles, and the material’s residence time in the muffle. Figure 6d,e show the formation of hexagonal structures and differently oriented agglomerated polyhedral shapes. The nanostructures’ morphology is attributed to the heat treatment and the effect of the chelating agent (ethylenediamine) [7,14,23,37]. According to the literature, applying chelating agents, such as ethylenediamine, in synthesizing materials like the one studied here favors the creation of organometallic complexes that form a template for particle growth. By calcining the MgSb_2_O_6_ powders, the organic material is eliminated, giving rise to octahedral and tetrahedral structures, nanorods, nanowires, and randomly shaped nanoparticles [25,26,28,37]. The nanoparticles’ size was estimated at 8.87–99.85 nm, with a mean of ~27.63 nm and a standard deviation of ~±17.69 nm (Figure 7). The calculation of the particles’ average size was carried out in areas where they were clearly visible.

### 3.5. Gas-Sensing Properties

**Figure 8:**
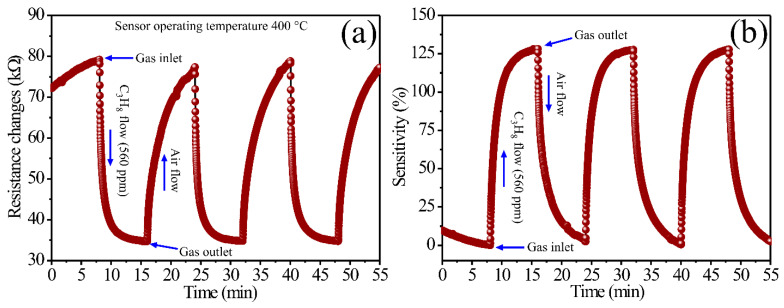
Dynamic response in C_3_H_8_ of the MgSb_2_O_6_ as a function of (**a**) the variation in electrical resistance, and (**b**) the dynamic sensitivity percentage.

**Figure 9:**
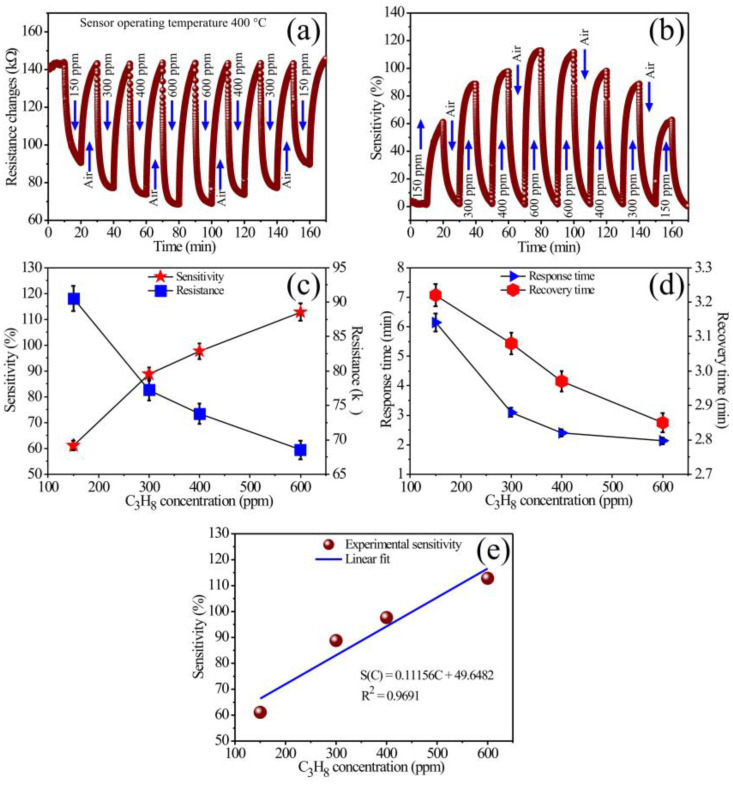
Dynamic response of the MgSb_2_O_6_ in C_3_H_8_ at 400 °C. (**a**) Variation of electrical resistance with gas concentration, (**b**) sensitivity vs. gas concentration, (**c**) variation of sensitivity and electrical resistance as a function of gas concentration, (**d**) response and recovery times of MgSb_2_O_6_ thick films, and (**e**) linear fitting of the response as a function of C_3_H_8_ concentration.

To evaluate the ability of the MgSb_2_O_6_ nanoparticles obtained at 700 °C to detect C_3_H_8_ atmospheres, experiments were carried out using, first, 560 ppm of the gas (Figure 8). Later, propane concentrations of 150, 300, 400, and 600 ppm were injected into the measuring chamber (Figure 9). In these tests, the MgSb_2_O_6_ films were always at 400 °C in a direct current (DC) of 200 μA. Films’ sensitivity was estimated with the formula $S=\left(R_{a}-R_{g}\right)/R_{g}$ × 100, where $R_{a}$ is air resistance and $R_{g}$ is test gas resistance. During the measurements, a constant flow of 1500 cm^3^/min of extra-dry air (21% O_2_, 79% N_2_) was employed to stabilize the thick films’ surface. For the 560 ppm sensing tests, the thick film surface was supersaturated by a flow of extra-dry air for the first 8 min. Then, the 560 ppm of C_3_H_8_ was injected for 8 min. Subsequently, the propane flow was stopped, observing that the thick films’ electrical resistance returned to its original value when the films were subjected to extra-dry air flows, thus corroborating the reproducibility of the material. This process was repeated cyclically until the test’s end (lasting approximately 55 min). The results are shown in Figure 8, where the change in electrical resistance (Figure 8a) and the sensitivity percentage (Figure 8b) at 400 °C can be observed.

As expected, when injecting the C_3_H_8_ at a constant current of 200 μA, the material’s electrical resistance decreased, while the sensitivity percentage increased considerably. The excellent dynamic response, the high sensitivity, and the good reproducibility shown by the MgSb_2_O_6_ films [14] were verified with the number of uniform cycles carried out during the test. The curves’ behavior shown in Figure 8a,b commonly occurs in an n-type semiconductor when exposed to atmospheres like the one studied here [2]. The variation in electrical resistance ranged from 34.22 to 79.74 kΩ, with an average of 45.52 kΩ. In contrast, the dynamic sensitivity range was 0.36 to 128.25%, with an average of 127.97%. To estimate the response and recovery times of the MgSb_2_O_6_ films, we considered 90% of the variation in electrical resistance in propane and 10% when exposed to air atmospheres [38]. Therefore, the calculated average response and recovery times were 1.898 and 5.63 min, respectively.

The excellent response shown in Figure 8 is mainly attributed to the chemical reactions between the test gas and the oxygen species ($O^{-}$ and $O^{2-}$-ionic forms [2,11]) previously adsorbed on the material’s surface due to the temperature (in our case, at 400 °C) [4]. These oxygen species are more reactive than those below 200 °C ($O_{2}^{-}$) [11]. It means that the C_3_H_8_ chemisorption on the film’s surface increased due to the temperature, favoring the mobility of the charge carriers (electrons) [14,23], which provoked variations in the electrical resistance and an increase in the material’s dynamic sensitivity. 

Figure 9 shows the dynamic tests’ results at different C_3_H_8_ concentrations (150, 300, 400, and 600 ppm) at constant temperature (400 °C) using a current of 200 μA. The experiments were carried out by injecting the propane concentrations into the measurement chamber, first increasing them (150–600 ppm) and then decreasing them (600–150 ppm). By varying the C_3_H_8_ concentrations, the MgSb_2_O_6_ films showed a drop in electrical resistance and increased dynamic sensitivity percentage as the gas concentration rose and vice versa (Figure 9a–c). With these experiments, we could test the reproducibility, stability, efficiency, and ability to detect low and high concentrations of C_3_H_8_. The average values of the electrical resistance in both directions for concentrations of 150, 300, 400, and 600 ppm were 52.50, 65.95, 68.90, and 70.59 kΩ, respectively. The respective sensitivity values were 61.09, 88.80, 97.65, and 112.81%. Using information from Kida et al. [38], we calculated the response and recovery times considering only the results for the increasing propane concentration (Figure 9d). The results are summarized in Table 1. 

On the other hand, calibration curves were obtained (Figure 9e) fitting to the equation S(C) = mC + b, where S is the response of the thick films, m is the response coefficient in C_3_H_8_, and b is power law’s constant. A correlation coefficient of 0.9691 indicates a good fit. This result suggests that a sensor made of MgSb_2_O_6_ can be employed for C_3_H_8_ concentrations within the range of 150 to 600 ppm, with 150 being the detection limit. 

Then, according to Figure 9, thick films’ response improved when the test gas concentration increased. This means that the increase in the material’s dynamic response was strongly related to the rise in the gas concentration, which reacted with the available oxygen ($O^{-}$) [11,15,17] on the films’ surface, causing greater charge carriers’ mobility due to the operating temperature’s effect [2,11] (400 °C). This increase in electrons’ kinetics was attributed to the rising concentration of the test gas, leading to stronger chemical reactions. This resulted in an increase in activation energy, enhancing electron mobility on the thick films’ surface due to the operating temperature [15,17]. This contributed to the observed variation in electrical resistance and sensitivity [14,20,23], significantly improving the material’s detection properties. It has been reported that such improvements in semiconductors allow an increase in their response if the test gas concentration is increased [15]. Wang et al. [39] and Ramírez-Ortega et al. [40] reported that the operating temperature is the most probable cause of the rise in the semiconductor’s response. Other studies report that the response depends on the test gas, its concentration, the material’s microstructure, and the operating temperature [5,6,11]. All these conditions favor a better diffusion of the gas molecules on the material’s surface, causing an increase in its response. Furthermore, the literature suggests that metallic oxides like ours exhibit faster response and recovery times due to the temperature effect and the high level of interaction between the thick films’ surface and the test gas, implying that the speed of oxygen adsorption and desorption increases, resulting in faster response and recovery times.

**Figure 10:**
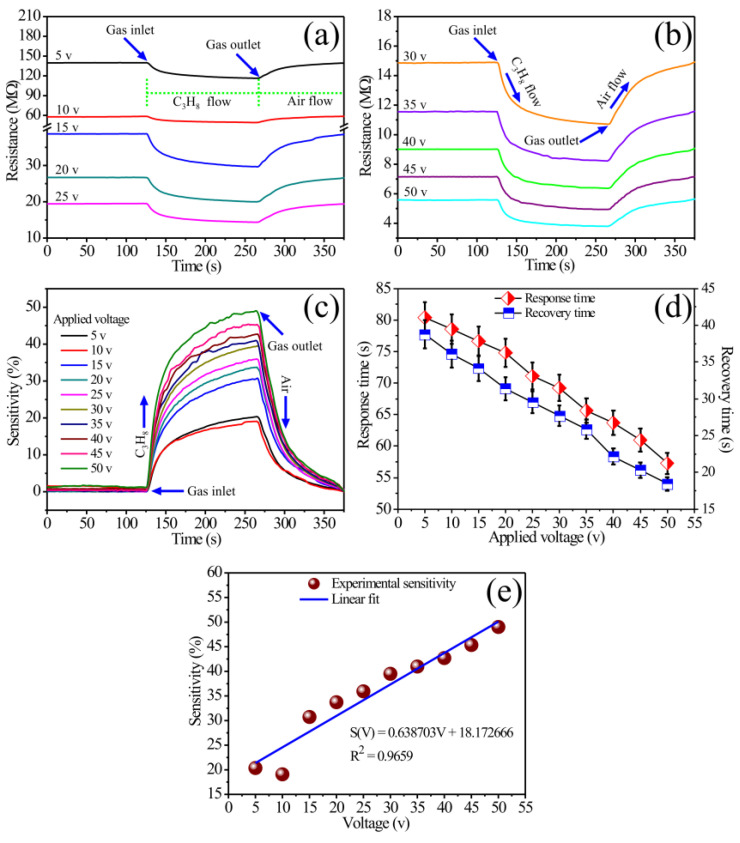
Dynamic response of the MgSb_2_O_6_ in C_3_H_8_ at 400 °C. (**a**,**b**) Variation in electrical resistance as a function of time, (**c**) dynamic sensitivity percentage as a function of time, (**d**) response and recovery times as a function of voltage, and (**e**) linear fitting of the response as a function of voltage.

Experiments were carried out at a concentration of 560 ppm at 400 °C to know the ability of the n-type MgSb_2_O_6_ to detect C_3_H_8_ atmospheres. A direct current (DC) signal was used for this, applying different voltages: 5, 10, 15, 20, 25, 30, 35, 40, 45, and 50 V. As for the results shown in Figure 8b and Figure 9b, sensitivity was calculated using the formula $S=\left(R_{a}-R_{g}\right)/R_{g}$ × 100, where $R_{a}$ is air resistance and $R_{g}$ is propane resistance. The results are shown in Figure 10. The data were graphed considering the variation in electrical resistance and the sensitivity percentage as a function of time (Figure 10a–c). A graph depicting the response and recovery times calculated as a function of the applied voltage (Figure 10d) is also shown. According to these results, the electrical resistance decreases with increasing voltage. This decrease in resistance is more obvious at higher voltages (30–50 V, Figure 10b). As can be observed, the rise in voltage and operating temperature favors the increase in energy, which causes greater mobility of the charge carriers on the material’s surface [40]. We observed that, when increasing the voltage at a constant temperature and constant flow of extra-dry air (21% O_2_), the thick films’ sensitivity percentage increased. This is associated with the enrichment of the oxygen species on the films’ surface due to the flow of extra-dry air, which caused a higher concentration of highly reactive oxygen species ($O^{-}$) [15,17] that reacted vigorously when the voltage and the temperature (400 °C) increased, favoring changes in the electrical resistance and, therefore, the increase in the material’s dynamic sensitivity percentage. We observed that with increasing operating temperature and voltage, oxidation of the test gas by oxygen species occurred, thus contributing to greater mobility of the charge carriers as well as an increase in the conductivity of the films [2,11], causing the material’s sensitivity to increase significantly [11]. For example, at 50 V, an increase in charge energy and an improvement in the oxygen adsorption and desorption on the surface are promoted, increasing the sensitivity of the MgSb_2_O_6_. According to the literature, the chemisorption of oxygen species as a function of temperature is the most likely cause of the films’ increase in dynamic response and sensitivity [13,14]. Additionally, our results indicate that increasing voltage also improves the material’s ability to detect C_3_H_8_ atmospheres.

The electrical resistance at 400 °C was 23.73, 9.34, 9.03, 6.67, 5.13, 4.18, 3.34, 2.65, 2.21, and 1.78 MΩ respective to the voltages 5, 10, 15, 20, 25, 30, 35, 40, 45, and 50 V. For the same voltages, the sensitivity percentages were 20.35, 19.09, 30.74, 33.72, 35.93, 39.50, 40.97, 42.71, 45.35, and 49.01%, respectively. The response and recovery times were calculated as in the previous case (according to reference [38]), considering 90% of the oxide’s electrical resistance variation in C_3_H_8_ and 10% in air. Thus, the response times respective to each voltage were 80.40, 78.53, 76.67, 74.83, 71.09, 69.24, 65.63, 63.70, 60.93, and 57.25 s. Recovery times were 38.77, 36.10, 34.15, 31.38, 29.54, 27.69, 25.85, 22.16, 20.31, and 18.45 s (see Table 2). Response and recovery times decreased considerably as the voltage increased while keeping the air flow and operating temperature constant. As previously mentioned, this is attributed to the fact that when the voltage changed, a vigorous chemical reaction (i.e., a high reaction rate) occurred between the test gas and the oxygen available on the thick films’ surface due to the temperature (400 °C) [40,41], causing a rapid response (i.e., a decrease in the material’s response and recovery times). 

From Figure 10e, it is evident that the sensor’s electrical response is correlated with the applied voltage and the optimal operating temperature that triggers the reaction of oxygen present on the sensor’s surface with the test gas at 400 °C. This correlation is supported by Figure 10a,b, which show the relationship between the decrease in electrical resistance and the increase in sensitivity percentage as a function of the applied voltage. A linear calibration curve corresponding to the sensor’s sensitivity percentage was plotted using the equation *S*(*V*) = *mV* + *b*, where *S* is the sensitivity, *m* is the sensitivity coefficient in propane, and b is the power law’s constant. The calculated R^2^ value of 0.9654 indicates a good fit, implying that the material can show a sensitivity percentage at voltages ranging from 5 to 50 V, consistently showing response increments as the voltage rises.

A possible chemical mechanism in propane detection for semiconductors like the one studied here has yet to be fully established. However, different authors have reported that the C_3_H_8_ detection process is effectively related to the operating temperature, the gas concentration, and the presence of oxygen [4,5,6]. When the material’s surface comes into contact with C_3_H_8_ molecules, the gas dissociates before reacting with oxygen, causing changes in the semiconductor’s electrical resistance [2,3,17]. When the propane molecules are adsorbed, they react with $O^{-}$ oxygen species on the surface, producing CO_2_, water vapor, and a release of electrons to the semiconductor material [7,14,42]. A possible chemical reaction that occurs in the detection of the C_3_H_8_ is the following [42]:

C_3_H_8_ + 10O_2_− → 3CO_2_ + 4H_2_O + e^−^

It means that when the propane is injected into the measurement chamber and comes into contact with the thick films’ surface, the gas molecules dissociate before reacting with the ionosorbed oxygen species [14,42], causing an electron transfer during the gas chemisorption [40,42]. This leads to a decrease in electrical resistance and an increase in the MgSb_2_O_6_’s sensitivity percentage. Another factor that significantly favors the excellent response of our oxide films is the use of the Sb cation in the synthesis process [29,31,32]. That is because by combining Sb^3+^ or Sb^5+^ with divalent cations such as Mg^2+^ (or Co^2+^, Zn^2+^, Ni^2+^), trirutile-type compounds (CoSb_2_O_6_, ZnSb_2_O_6_, NiSb_2_O_6_, respectively) are produced, which are thermally stable when exposed to toxic atmospheres [22,26,40]. It is reported in the literature that an advantage of using materials composed of the Sb cation (such as the trirutile-types) is that they substantially improve the catalytic activity and, with it, the ability to detect gases like CO, CO_2_, liquefied petroleum gas (LPG), and propane [14,22,26], among others. Also, a high chemical reactivity is favored for semiconductors containing Sb, which improves their catalytic properties (see Table 3) [7,14,22,29]. This offers a tremendous advantage for detecting toxic (CO, CO_2_) and flammable (C_3_H_8_) gases compared to other semiconductor oxides that have been studied as gas sensors [26,28,42]. 

For instance, in a study [14], it was found that ZnAl_2_O_4_ took 176 s to respond and 205 s to recover when exposed to 1000 ppm of propane. Another study [22] found that CoSb_2_O_6_ took 21 s to respond and 234 s to recover when exposed to 5000 ppm of propane. Lastly, MgSb_2_O_6_ showed a response when exposed to 500 ppm of propane [28]. In this work, we found that MgSb_2_O_6_ had a response time of 57.2 s and a recovery time of 18.5 s when exposed to low concentrations of propane gas (560 ppm). So, our results suggest that MgSb_2_O_6_ can be considered an excellent gas sensor.

In summary, using Sb and Mg cations to form the trirutile-like structure of the MgSb_2_O_6_ contributed to improving its catalytic activity and the diffusion of the test gas on the thick films’ surface at 400 °C. It was verified that the excellent compound’s detection ability, reproducibility, and efficiency are mainly due to the microstructural characteristics and the nanometric particle size (in our case, ~27.63 nm). Additionally, some studies suggest that oxygen adsorption and desorption, as well as the microstructure of the material, are additional factors that affect the detection properties of the sensor [4,11,15]. Specifically, if the particle size is less than half the thickness of the outer layer (LS) [17,27], then the adsorbed oxygen species are responsible for the variation in detection properties and an increase in sensitivity. Furthermore, if the crystal or particle size is D < 2L, the crystals participate in electronic transport during sensing [17,27]. This leads to variations in electrical resistance, resulting in an increase in the sensor’s response, high electrical sensitivity, thermal stability, and high efficiency. By reducing the MgSb_2_O_6_ particles’ dimensions, their surface area increased, thus improving the diffusion of C_3_H_8_ on the films’ surface due to the operating temperature. This caused the oxygen adsorption and desorption process to improve on the surface and, with it, changes in the material’s conductivity (or electrical resistance) [20,23,26]. In agreement with the literature, the improvement of the gas-sensing ability of our material, as well as its thermal stability and electrical response, occurred when its particle size was brought to a nanometric scale [2,3,11]. Similarly, with the use of the Sb cation in preparing the MgSb_2_O_6_, its response and recovery times, as well as the dynamic response, were optimized. This can be corroborated by the results shown in Figure 8, Figure 9 and Figure 10. 

## 4. Conclusions

The synthesis of MgSb_2_O_6_ nanoparticles was achieved using a chemical method aided by microwave radiation, resulting in a crystalline phase without secondary phases after heating at 700 °C for five hours. SEM analysis revealed polyhedral and bar-shaped morphologies, and TEM estimated the particle size to be 8.87–99.85 nm, with a mean of ~27.63 nm and a standard deviation of ±17.69 nm. The material’s band gap was determined to be ~3.86 eV using UV-Vis spectroscopy. Dynamic detection tests in propane atmospheres revealed a good response of the oxide at various gas concentrations (150, 300, 400, and 600 ppm) and operating voltages (5–50 V) with a maximum sensitivity of ~112.81 at 600 ppm and 400 °C. The material also showed a sensitivity of ~61.09 at lower gas concentrations (150 ppm) with response and recovery times of 6.15 and 3.22 min. These excellent results can be attributed to the synthesis method, operating temperature, and the material’s microstructure (bars and polyhedrons). Therefore, MgSb_2_O_6_ shows excellent potential as a propane sensor at concentrations as low as 150 ppm.

## Figures and Tables

**Table 1:** Mgsb_2_O_6_ thick films at different C_3_H_8_ concentrations.

Concentration (ppm)	ΔR (kΩ)	Sensitivity (%)	Response Time (min)	Recovery Time (min)
150	52.50	61.09	6.15	3.22
300	65.95	88.80	3.10	3.08
400	68.90	97.65	2.40	2.97
600	70.59	112.81	2.13	2.85

**Table 2:** Variation of electrical resistance, sensitivity, and response and recovery times of the MgSb_2_O_6_ sensor at different operating voltages.

Voltage (V)	ΔR (MΩ)	Sensitivity (%)	Response Time (s)	Recovery Time (s)
5	23.73	20.35	80.40	38.77
10	9.34	19.09	78.53	36.10
15	9.03	30.74	76.67	34.15
20	6.67	33.72	74.83	31.38
25	5.13	35.93	71.09	29.54
30	4.18	39.50	69.24	27.69
35	3.34	40.97	65.63	25.85
40	2.65	42.71	63.70	22.16
45	2.21	45.35	60.93	20.31
50	1.78	49.01	57.25	18.45

**Table 3:** Performance comparison of propane sensors based on metal oxides.

Material	Gas	Concentration (ppm)	Sensitivity (%)	Response Time (s)	Recovery Time (s)	Reference
ZnAl_2_O_4_	Propane	1000 ppm	-	176.0	205.0	[14]
CoSb_2_O_6_	LPG	5000 ppm	1.96	21.0	234.0	[22]
ZnSb_2_O_6_	LPG	5000 ppm	1.73	41.0	95.0	[26]
MgSb_2_O_6_	Propane	500 ppm	61.66	-	-	[28]
IrO_2_	-	1000 ppm	-	-	-	[42]
MgSb_2_O_6_ MgSb_2_O_6_	Propane Propane	600 ppm 560 ppm	112.81 49.01	127.8 57.2	171 18.5	This work This work

## Data Availability

The data that support the findings of this study are available from the corresponding author upon request.
